# Unsteady Pressure-Driven Electrokinetic Slip Flow and Heat Transfer of Power-Law Fluid through a Microannulus

**DOI:** 10.3390/mi14020371

**Published:** 2023-02-01

**Authors:** Shuyan Deng, Ruiqing Bian, Jiacheng Liang

**Affiliations:** 1 Institute of Architecture and Civil Engineering, Guangdong University of Petrochemical Technology, Maoming 525000, China; 2 College of Civil Engineering, Dalian Minzu University, Dalian 116600, China

**Keywords:** power-law fluids, electrokinetic flow, unsteady velocity, heat transfer, slip length

## Abstract

To guarantee the transporting efficiency of microdevices associated with fluid transportation, mixing, or separation and to promote the heat transfer performance of heat exchangers in microelectronics, the hydrodynamic behaviors at unsteady and steady states, as well as the thermal characteristics at the steady state in a pressure-driven electrokinetic slip flow of power-law fluid in a microannulus are studied. To present a more reliable prediction, the slip phenomenon at walls and nonlinear rheology of liquid are incorporated. The modified Cauchy momentum equation applicable to all time scales and energy equations, are analytically solved in the limiting case of a Newtonian fluid and numerically solved for power-law fluids. The transient velocity profile, time evolution of flow rate, temperature profile, and heat transfer rate are computed at different flow behavior indices, electrokinetic width, slip lengths, and Brinkman numbers, thereby, the coupling effect of nonlinear rheology, slip hydrodynamics, and annular geometry on flow and thermal behaviors is explored. The unsteady flow takes a longer time to achieve the steady state for shear thinning fluids or greater slip lengths. The flow behavior index and slip length play a significant role in the flow rate and heat transfer performance. The relevant discussion can serve as a theoretical guide for the operation and thermal management of annular geometry-related flow actuation systems.

## 1. Introduction

In microflows, the surface effect predominates, and the contact between the charged surface of the channel wall and ionic liquid leads to the redistribution of nearby ions, inducing the electric double layer (EDL) [1]. The application of an external electric field tangential to the liquid in the microchannel results in the migration of excess counter ions in the EDL, finally leading to electroosmotic flow (EOF) under the viscous drag force, which is generally termed electrokinetic flow. With the development of the micro-electronic-mechanical system (MEMS), and due to its desirable attributes, electroosmosis is used as a flow actuation mechanism in chemical and biomedical analysis, membrane separation, soil remediation, and the thermal management of microelectronic systems [2,3].

To meet the growing demand for electroosmosis actuation systems, a vast majority of the literature has been reported from different aspects, such as the influence of different geometries on EOF and theoretical surveys on two-layer electroosmosis systems [4,5,6,7]. Among them, the electrokinetic flow in an annular geometry remains an extensive topic of scientific and technological interest. From a scientific point of view, the cylindrical (rectangular) annulus model is of more universal significance, and can be treated as a parallel plate or circular cylinder (rectangular microchannel) in the limiting cases of the ratio of the inner radius to the outer radius [8,9], and can also serve as a basis for the development of two-layer fluid systems [9,10]. From a practical point of view, in studying the chemical remediation of contaminated soil, treating the pores inside the soils as microannulus rather than cylinders can provide a more reliable prediction for the flow rate of an electrokinetic flow [11]. The problem formulation of the EOF through an annulus involves modeling the electrokinetic motion of a particle in a polymeric porous membrane, thus, it is significant to the investigation of the electrophoretic separation of proteins [12]. In this context, Tsao analyzed the hydrodynamical behavior in a steady EOF through a microannulus [13], which was extended to that under high zeta potentials by Kang et al. [8]. Furthermore, due to the frequent application of time-periodic electroosmosis in electrokinetic instability and simulation of human processes, the flow behavior of time-periodic EOF at the steady state was investigated in [14]. The model above was extended to that applicable to all time scales and solved by Moghadam using Green’s function method [15] and by Wang et al. by means of the integral transform method [16], separately, thereby the time-periodic flow behavior at the unsteady state was examined. In addition, Chang et al. investigated the unsteady EOF through an annulus under a low zeta potential [17] and arbitrary zeta potential assumptions [18] by presenting an analytical series solution of velocity distribution.

The literature above made an effort to extend the basic knowledge of hydrodynamical aspects in Newtonian fluid flow through annular geometry. However, the operation of biofluids, DNA solution, and colloidal suspensions is common in drug delivery systems and Lab-on-chip, and those fluids show non-Newtonian rheological behaviors by nature [19]. The power-law model was studied by Das and Chakraborty to analytically investigate the EOF of blood [20], which was then extended by numerous researchers because of its concise expression and wide coverage. Annular geometry was also adopted as a novel microfluidic mode in the blending of chemicals or biofluids [12], which can be viewed as hollow microneedles and used for the extraction of body fluids (blood and saliva) and the accurate dispensing of liquids [15]. Unfortunately, the study on the EOF problems of a non-Newtonian fluid through a microannulus is limited. A theoretical analysis was carried out for the mass transfer in oscillatory EOF of Maxwell fluids and it turns out that the velocity and concentration distributions depend notably on the elasticity number of the Maxwell fluid [21]. The time-periodic EOF of Jeffreys fluids was investigated by using the Laplace transform method and the velocity for different relaxation times and annular radius ratios was presented [22].

Microfluidics such as Lab-on-chip deals with thermally liable samples, and thus temperature variation, might lead to the low column efficiency and reduction in analysis resolution as well as the loss of injected samples [23]. Therefore, another important category of the research on the electroosmosis problem is thermal characteristics [24,25,26,27]. Due to the large heat transfer area and high heat transfer coefficient, annular geometry is frequently encountered in micro heat exchangers and microelectronic cooling [28,29]. Nevertheless, only some literature reported the exploration of the thermal behaviors of electroosmosis in an annulus. The thermal effects in a mixed electroosmotic pressure-driven flow of power-law fluids were numerically investigated in [30]. Yavari et al. numerically analyzed both the hydrodynamic and thermal characteristics of an electrokinetic flow in the absence of viscous dissipation [28]. The numerical results show that the annular geometry parameter exerts an impressive influence on the heat transfer performance. In addition, Moghadam explored the thermal behaviors of AC EOF for different sorts of constant wall heat fluxes [31].

All the aforementioned literature adopted no-slip boundary conditions at walls, however, which is an empirical model lacking a solid theoretical basis. Recent experiments show that fluids slip over the surface [32,33,34], therefore, the velocity is inevitably higher than that without consideration of the slip condition. The steady mixed electroosmotic pressure-driven flow of power-law fluids in slit microchannels was investigated with consideration of the slip condition by presenting semi-analytical and numerical solutions [35]. It is common in microfluidics that the microchannels through which electrokinetic flow occurs are fabricated from different materials such as poly-dimeethysiloxane (PDMS) showing hydrophobic properties. Therefore, the different electroosmosis actuation systems, including single layer and two layer, in hydrophobic microchannel considering slip conditions, were separately studied in [34] and [36].

To the best of the authors’ knowledge, there is still a lack of comprehensive investigation on the unsteady slippery EOF of power-law fluids through an annulus. However, the corresponding analysis not only reveals the underlying mechanism that how the fluid is initiated but is also of practical use in the operation of bio-MEMS and remediation of contaminated soil. And the search for effective ways to drive various fluids into microchannels of different geometries persists. Bearing this in mind, this paper aims to explore the slip hydrodynamics at unsteady and steady states, as well as thermal characteristics in the pressure-driven electrokinetic flow of power-law fluids through a microannulus. The fundamental understanding of this phenomenon is crucial for guaranteeing the transporting efficiency of microdevices associated with fluid transportation, mixing or separation, and promoting the heat transfer performance of heat exchangers in microelectronics. For the sake of generality, the flow of the power-law fluid is driven by the pressure gradient and electric field. In addition, the slip phenomenon at the walls is incorporated in mathematical formulation to obtain a more realistic microflow model. The novelty of the present work is the investigation of the flow mechanism for all time scales under the nonlinear coupling of power-law rheology, slippery interfacial hydrodynamics, and annular geometrical effect, as well as the evaluation of the heat transfer performance of a slip flow system under the combined effect of viscous dissipation and Joule heating. The governing equations are analytically solved in the limiting case of a Newtonian fluid and are numerically solved for power-law fluids. The velocity and temperature distributions, flow rate, and heat transfer rate are evaluated at different pertinent parameters.

## 2. Mathematical Modeling

### 2.1. Modified Cauchy Momentum Equation

The continuity equation and Cauchy momentum equation for an incompressible laminar flow are given as [4].

**Figure 1:**
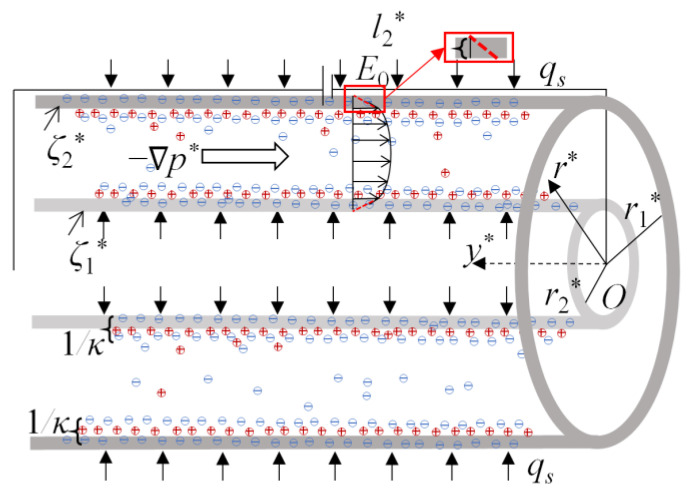
The schematic of the pressure-driven electrokinetic slip flow in a cylindrical microannulus.

(1) ∇⋅V=0

(2) $$\rho [\frac{\partial V}{\partial t}+(V\cdot \nabla )V]=\nabla \cdot \tau +F-\nabla p^{*}$$

where V is the velocity vector, p^*^ is the pressure, τ is the stress for power-law fluid, F is the body force acting on liquid, and *ρ* is the liquid density. Here, the flow is driven under the combined effect of electroosmotic force arising from the applied electric field, pressure gradient, and slip hydrodynamics at the walls, as sketched in Figure 1. The microannulus is characterized by the inner radius *r*_1_^*^ and outer radius *r*_2_^*^ in which the slip lengths near the inner and outer walls are l^*^_1_ and l^*^_2_, respectively, and the strength of the electric field is *E*_0_. To facilitate the analysis and capture the primary physics of slip flow, the following assumptions are applied: Equation (1) the thickness of EDLs at two walls is far less than r_i_^*^, and thus the EDLs will not overlap, the zeta potentials near the inner and outer walls ζ_i_^*^ are constant; Equation (2) in microchannels, the gravity of fluid is negligible; Equation (3) since the length of the microchannel is far longer than the characteristic radius of microannulus, the radial velocity can be ignored and only the axial velocity v^*^ is considered; and Equation (4) the slip phenomenon is represented by Navier’s slip model [36]. Applying the assumptions above to Equations (1) and (2), one has the modified Cauchy momentum equation, the slip conditions at the channel walls, and the initial condition [36,37].

(3) $$\frac{\partial v^{*}}{\partial t^{*}}=\frac{1}{r^{*}}\frac{\partial }{\partial r^{*}}\left(r^{*}\frac{\eta^{*}}{\rho }\frac{\partial v^{*}}{\partial r^{*}}\right)+\frac{1}{\rho }\rho_{e}E_{0}^{*}-\frac{1}{\rho }\nabla p^{*}$$

(4) $$v^{*}{|}_{r^{*}=r_{1}^{*}}-l_{1}^{*}\frac{\partial v^{*}}{\partial r^{*}}=0,\;v^{*}{|}_{r^{*}=r_{2}^{*}}+l_{2}^{*}\frac{\partial v^{*}}{\partial r^{*}}=0,\;v^{*}{|}_{t^{*}=0}=0$$

In Equation (3), η^*^ = *m_n_*|∂v^*^/∂r^*^|*^n^*^−1^ represents the apparent viscosity of the power-law fluid where *n* denotes the flow behavior index of the unit [1], *n* < 1, *n* = 0, and *n* > 1 physically represent the shear thinning, Newtonian, and shear thickening fluids, respectively, and *m_n_* denotes the flow consistency index of the unit [Nm^−2^s*^n^*] which reduces to the dynamic viscosity of the Newtonian fluid, namely, *m*_0_ of the unit [Nm^−2^s], when *n* = 1. In Equation (3), the local net charge density is subject to Boltzmann distribution based on the assumption of a local thermodynamic equilibrium *ρ_e_* = −2*ezn*_0_ sinh[(ezψ^*^)/(*k_B_T_a_*)]. The electric potential distribution ψ^*^ for a symmetric electrolyte due to the presence of the EDL is determined by the well-known Poisson–Boltzmann (P–B) equation [1,4].

(5) $$\nabla^{2}\psi^{*}=\frac{2ezn_{0}}{\varepsilon }\sinh(\frac{ez\psi^{*}}{k_{B}T_{a}})$$

where *e* denotes the elementary charge, *z* denotes the ion valence, *n*_0_ is the ion concentration in liquid, *k_B_* is the Boltzmann number, and *T_a_* is the absolute temperature. In Equation (4), l^*^_1_ and l^*^_2_ are the property parameters of the solid wall and working liquid, representing the constant slip lengths near the inner and outer walls, respectively. Physically, l^*^_i_ indicates the thickness beyond the solid-liquid interface where the velocity extrapolates to zero, as shown in Figure 1. According to the Debye–Hückel linear approximation [1], the hyperbolic term sinh[(ezψ^*^)/(*k_B_T_a_*)] can be linearized as [(ezψ^*^)/(*k_B_T_a_*)] by expanding the hyperbolic function up to first order, and it can work well when [(ezψ^*^)/(*k_B_T_a_*)] < 1, physically for small zeta potentials, as compared to the thermal energy of ions, *k_B_T_a_*/*e* < 25.7 mV [4]. Consequently, the electric potential distribution is governed by the following linearized P–B equation [15].

(6) $$\frac{1}{r^{*}}\frac{d}{dr^{*}}\left(r^{*}\frac{d\psi^{*}}{dr^{*}}\right)=\frac{2zen_{0}}{\varepsilon }\frac{ze\psi^{*}}{k_{B}T_{a}}$$

which is subject to the boundary conditions [15]

(7) $${\left.\psi^{*}\right|}_{r^{*}=r_{1}^{*}}=\zeta_{1}^{*},\;{\left.\psi^{*}\right|}_{r^{*}=r_{2}^{*}}=\zeta_{2}^{*}$$

Introducing the dimensionless variables *r* = r^*^/*R*, ψ^*^ = zeψ^*^/(*k_B_T_a_*), *v* = v^*^/*V_hs,_* and *t* = t^*^m_0_/(*ρR*^2^) to Equations (3), (4), (6) and (7) produces the dimensionless governing equations [36,37].

(8) $$\frac{\partial v}{\partial t}=\alpha \frac{1}{r}\frac{\partial }{\partial r}\left(r{\left|\frac{\partial v}{\partial r}\right|}^{n-1}\frac{\partial v}{\partial r}\right)-W^{2}\psi -\nabla p$$

(9) $$\left(v-l_{1}\frac{\partial v}{\partial r}\right){|}_{r=r_{1}}=0,\;\left(v+l_{2}\frac{\partial v}{\partial r}\right){|}_{r=r_{2}^{}}=0,\;v{|}_{t=0}=0$$

(10) $$\frac{d^{2}\psi }{dr^{2}}+\frac{1}{r}\left(\frac{d\psi }{dr}\right)-W^{2}\psi =0$$

(11) $${\left.\psi \right|}_{r=r_{1}}=\zeta_{1}^{},\;{\left.\psi \right|}_{r=r_{2}}=\zeta_{2}^{}$$

in which *α* = *m_n_*(*V_hs_*/*R*)*^n^*^−1^/*m*_0_, ∇*p* = ∇p^*^V_hs_m_0_/(*ρR*^2^), and *r_i_* = r^*^_i_/*R* with *i* = 1,2, and *V_hs_* = *ɛk_B_T_a_E*_0_/(*ezm*_0_) means the Helmholtz–Smoluchowski electroosmotic velocity. *W* = *κR* is the electrokinetic width with 1/*κ* = [2*e*^2^*z*^2^*n*_0_/(*εk_B_T_a_*)]^−1/2^, denoting the thickness of EDLs, and *R* is the reference radius, where *l_i_* = l^*^_i_/*R* represents the dimensionless slip length, and *ζ_i_* represents the dimensionless zeta potential where the subscripts *i* = 1 and *i* = 2 stand for the inner and outer walls, respectively.

Using Equations (8) and (9), one has the dimensionless unsteady flow rate

(12) $$Q(t)=2\pi \int_{r_{1}}^{r_{2}}vrdr$$

### 2.2. Energy Equation

The temperature distribution T^*^ for the thermally fully developed steady flow is governed by the energy equation [26]

(13) $$(\rho c_{p})v_{s}^{*}\frac{\partial T^{*}}{\partial z^{*}}=k\frac{1}{r^{*}}\frac{\partial }{\partial r^{*}}\left(r^{*}\frac{\partial T^{*}}{\partial r^{*}}\right)+\sigma E_{0}^{2}+\eta {\left(\frac{dv_{s}^{*}}{dr^{*}}\right)}^{2}$$

(14) $$q_{s}=k\frac{\partial T^{*}}{\partial r^{*}}{|}_{r^{*}=r_{1}^{*}}\;(T^{*}{|}_{r^{*}=r_{1}^{*}}=T_{w}^{*}),\;q_{s}=k\frac{\partial T^{*}}{\partial r^{*}}{|}_{r^{*}=r_{2}^{*}}\;(T^{*}{|}_{r^{*}=r_{2}^{*}}=T_{w}^{*})$$

where *c_p_* denotes the specific heat at constant pressure, *k* is the thermal conductivity, *σ* is the electric conductivity of the liquid, v_s_^*^ is the velocity distribution of steady flow, *q_s_* denotes the constant wall heat flux, and T^*^_w_ denotes the wall temperature. Further, since the constant heat flux boundary condition *q_s_* = *const* is adopted, one has *∂*[(T^*^_w_ − T^*^)/(T^*^_w_ − T^*^_m_)]/∂r^*^ = 0, thereby, ∂T^*^/∂r^*^ = dT^*^_w_/dr^*^ = dT^*^_m_/dr^*^≡*const* in which T^*^_m_ denotes the mean temperature. Imposing the overall energy balance condition over an elemental control volume yields [26]

(15) $$\frac{dT_{m}^{*}}{dr^{*}}=\frac{\left[2\pi r_{1}^{*}q_{s}+2\pi r_{2}^{*}q_{s}+\sigma E_{0}{}^{2}\pi (r_{2}^{*2}-r_{1}^{*2})+\eta \int_{r_{1}^{*}}^{r_{2}^{*}}{\left(\frac{\partial v_{s1}^{*}}{\partial r_{}^{*}}\right)}^{2}2\pi r_{}^{*}dr^{*}\right]}{\left(\rho c_{p})v_{sm}^{*}\pi (r_{2}^{*2}-r_{1}^{*2}\right)}$$

where v^*^_sm_ indicates the mean temperature of steady flow. Introducing the dimensionless group *T* = *k*(T^*^ − T^*^_w_)/(*q_s_R*), *v_s_* = v_s_^*^/*V_hs_*, $F_{1}=\int_{r_{1}}^{r_{2}}v_{s}rdr$, $F_{2}=\int_{r_{1}}^{r_{2}}{\left|\partial v_{s}^{}/\partial r\right|}^{n-1}{\left(\partial v_{s}^{}/\partial r\right)}^{2}rdr$, $S=\sigma E_{0}^{2}R/q_{s}$ and $Br=m_{n}V_{hs}^{2}/(q_{s}R){\left(V_{hs}^{}/R\right)}^{n-1}$ into Equations (13) and (14), and combining with Equation (15), one has the dimensionless energy equations

(16) $$\frac{1}{r}\frac{d}{dr}\left(r\frac{dT}{dr}\right)-\frac{r_{2}+r_{1}+S(r_{2}^{2}-r_{1}^{2})/2+BrF_{2}}{F_{1}}v_{s}+S+Br{\left|\frac{dv_{s}^{}}{dr}\right|}^{n-1}{\left(\frac{dv_{s}^{}}{dr}\right)}^{2}=0$$

(17) $$T{|}_{r=r_{1}^{}}=0,T{|}_{r=r_{2}^{}}=0$$

where the first term in the left-hand side (LHS) of Equation (16) denotes the heat generation caused by heat diffusion, and the remaining terms in LHS denote the heat generations arising from axial conduction, Joule heating, and viscous dissipation, respectively. To note, *S* is the Joule heating parameter and *Br* is the Brinkman number, representing the viscous dissipation effect. To provide an in-depth insight into the heat transfer performance of electrokinetic slip flow, the first law analysis is conducted, i.e., the Nusselt number implying the heat transfer efficiency is deduced [26,38]. According to *Nu* = 2*q_s_*(r^*^_2_ − r^*^_1_)/(T^*^_w_ − T^*^_m_)/*k* and the mean temperature

(18) $$T_{m}=k(T_{m}^{*}-T_{w}^{*})/(q_{s}R)=\int_{r_{1}}^{r_{2}}Tv_{s}rdr/\int_{r_{1}}^{r_{2}}v_{s}rdr$$

one has the Nusselt number for the slip flow in a microannulus

(19) $$Nu=-\frac{2(r_{2}-r_{1})}{T_{m}}$$

## 3. Solution Method and Validation

First, P–B Equations (10) and (11) are solved

(20) $$\psi (r)=AI_{0}(Wr)+BK_{0}(Wr)$$

where *I*_0_ and *K*_0_ imply the 0-th order modified Bessel functions of the first kind and second kind, respectively, and the coefficients are given as $A=\frac{\zeta_{2}K_{0}(Wr_{1})-\zeta_{1}K_{0}(Wr_{2})}{K_{0}(Wr_{1})I_{0}(Wr_{2})-K_{0}(Wr_{2})I_{0}(Wr_{1})}$ and $B=\frac{\zeta_{2}I_{0}(Wr_{1})-\zeta_{1}I_{0}(Wr_{2})}{K_{0}(Wr_{2})I_{0}(Wr_{1})-K_{0}(Wr_{1})I_{0}(Wr_{2})}$.

### 3.1. For Newtonian Fluid (n = 1) and without Consideration of Viscous Dissipation (Br = 0)

In the limiting case of a Newtonian fluid, Equations (8) and (9) reduce to

(21) $$\frac{\partial v^{N}}{\partial t}=\frac{\partial^{2}v^{N}}{\partial r^{2}}+\frac{1}{r}\frac{\partial v^{N}}{\partial r}-W^{2}\psi -\nabla p$$

(22) $$\left(v^{N}-l_{1}\frac{\partial v^{N}}{\partial r}\right){|}_{r=r_{1}}=0,\;\left(v^{N}+l_{2}\frac{\partial v^{N}}{\partial r}\right){|}_{r=r_{2}^{}}=0,v^{N}{|}_{t=0}=0$$

where *v^N^* is the velocity of a Newtonian fluid. Since it is inhomogeneous, Equation (21) is homogenized as

(23) $$\frac{\partial {\tilde{v}}^{N}}{\partial \tilde{t}}=\frac{\partial^{2}{\tilde{v}}^{N}}{\partial r^{2}}+\frac{1}{r}\frac{\partial {\tilde{v}}^{N}}{\partial r}$$

(24) $$\left({\tilde{v}}^{N}-l_{1}\frac{\partial {\tilde{v}}^{N}}{\partial r}\right){|}_{r=r_{1}}=0,\left({\tilde{v}}^{N}+l_{2}\frac{\partial {\tilde{v}}^{N}}{\partial r}\right){|}_{r=r_{2}^{}}=0,{\tilde{v}}^{N}{|}_{\tilde{t}=t-\tau =0}=-W^{2}\psi -\nabla p$$

Using the method of variable separation, Equations (23) and (24) are solved to

(25) $${\tilde{v}}^{N}(r,\tilde{t};\tau )=\sum_{m=1}^{\infty }C_{m}M_{0}(\lambda_{m}r)e^{-\lambda_{m}^{2}\tilde{t}}$$

The coefficient *C_m_* and the basis function *M*_0_(*λ_m_r*) are presented in the Appendix A for conciseness and readability. Applying Duhamel’s principle and combining Equation (25), the solution to Equations (21) and (22) is obtained as

(26) $$v^{N}=\int_{0}^{t}{\tilde{v}}^{N}(r,\tilde{t};\tau )d\tau =\sum_{m=1}^{\infty }\frac{C_{m}}{\lambda_{m}^{2}}M_{0}(\lambda_{m}r)-\sum_{m=1}^{\infty }\frac{C_{m}}{\lambda_{m}^{2}}M_{0}(\lambda_{m}r)e^{-\lambda_{m}^{2}t}$$

As the slip flow evolves to the steady state, the temporal term in Equation (21) vanishes and the steady velocity is solved from Equations (21) and (22) as

(27) $$v_{s}^{N}=\psi +\frac{1}{4}\nabla pr^{2}+D\ln r+E$$

where the coefficients *D* and *E* are provided in the Appendix A. The first and second terms in the RHS of Equation (26) represent the steady velocity and the transient one, respectively. When *t*→∞, the second term in Equation (26) vanishes and thus, *v^N^*→*v_s_^N^*. Therefore, in evaluating the unsteady velocity, to eliminate the oscillation caused by a truncated error in series solution (26), the first term in Equation (26) is replaced with *v_s_^N^*, given by Equation (27), and the final version of velocity for unsteady slip flow reads

(28) $$v_{}^{N}=\left(\psi +\frac{1}{4}\nabla pr^{2}+D\ln r+E\right)-\sum_{m=1}^{\infty }\frac{C_{m}}{\lambda_{m}^{2}}M_{0}(\lambda_{m}r)e^{-\lambda_{m}^{2}t}$$

Similarly, as *t*→∞, *v^N^* automatically becomes *v_s_^N^*. With *v_s_^N^* obtained, for the Newtonian fluid, Equations (16) and (17) are analytically solved in the absence of viscous dissipation (*Br* = 0)

(29) $$T_{}^{N}=\frac{F}{W^{2}}\psi +\frac{F\nabla p}{64}r^{4}+\frac{1}{4}(EF-S-DF)r^{2}+\frac{DF}{4}r^{2}\ln r+\left[C_{0}-Q_{0}(r_{1})\right]\ln r+T_{0}$$

where the coefficients *F*, *C*_0_, *T*_0_, and the intermediate function *Q*_0_(*r*) are presented in the Appendix A. The symbolic simplification of several coefficients is carried out with the help of Maple, and the computation and graphical presentation of Equations (28) and (29) are carried out using Matlab.

### 3.2. For Power-Law Fluids (n ≠ 1) and with Consideration of Viscous Dissipation (Br ≠ 0)

For power-law fluids and when considering the viscous dissipation effect, the momentum Equations (8) and (9) and the energy Equations (16) and (17) are solved using the finite difference method. Let *t_l_* = *l*Δ*t*, *r_j_* = *j*Δ*r*, *v^l^_s_*_,*j*_ = *v_s_*(*j*Δ*r*,*l*Δ*t*), *v^l^_j_* = *v*(*j*Δ*r*,*l*Δ*t*), *ψ_j_* = *ψ*(*j*Δ*r*), and *T^l^_j_* = *T*(*j*Δ*r*,*l*Δ*t*), *l* = 1,2,…,*L* and *j* = 1,2,…,*J*. The explicit finite difference scheme is used for time and the central difference scheme is adopted for space. According to Equation (9), the numerical velocity at the boundaries is *v^l^*_1_ = *l*_1_(4*v^l^*_2_ – *v^l^*_3_)/(2Δ*r +* 3*l*_1_) and *v^l^_J_* = *l*_2_(4*v^l^_J_*_−1_ – *v^l^_J_*_−2_)/(2Δ*r +* 3*l*_2_). The bulk liquid velocity is computed by following the numerical algorithm

(30) $$v_{j}^{l+1}=v_{j}^{l}+\Delta t[\Pi_{j}^{l}-W^{2}\psi_{j}-\nabla p]$$

$\Pi_{j}^{l}=\alpha {\left(g_{j}^{l}\right)}^{n-1}\left(\frac{v_{j+1}^{l}-v_{j-1}^{l}}{2r_{j}\Delta r}+\frac{v_{j+1}^{l}-2v_{j}^{l}+v_{j-1}^{l}}{\Delta r^{2}}\right)+\alpha (n-1){\left(g_{j}^{l}\right)}^{n-2}\frac{g_{j+1}^{l}-g_{j-1}^{l}}{2\Delta r}\frac{v_{j+1}^{l}-v_{j-1}^{l}}{2\Delta r}$. $g_{j}^{l}=\left|\frac{v_{j+1}^{l}-v_{j-1}^{l}}{2\Delta r}\right|$, *j* = 2,3,…,*J* – 1. When *t→*∞, the transient velocity approaches steady velocity *v^l^_s_*, i.e., $|v^{l}-v^{l+1}|<err$ with *err* being a specified criterion. Consequently, the flow rates can be computed from Equation (12) by the composite trapezoidal integration method.

The temporal term *∂T*/*∂t* has been introduced in the RHS of Equation (16), which vanishes when *t*→∞, and *T* becomes the fully developed temperature. The numerical velocity and temperature are given as vectors $V^{l}={\left[v_{s,j}^{l}\right]}_{1\times J}^{T}$ and $T^{l}={\left[T_{j}^{l}\right]}_{1\times J}^{T}$. The Crank–Nicolson scheme is used for time and the central difference scheme is adopted for space. Combining with the boundary conditions (17), the numerical algorithm is

(31) $${Τ}^{l+1}{=\Lambda }^{-1}{\Gamma Τ}^{l}{+\Lambda }^{-1}\Psi \times \Delta t$$

Here, Λ = *tridiag*(I,A,I) + *sparse*(2,1,−*a* + *b*,*J*,*J*) + *sparse*(*J* − 1,*J*,−*a* − *b*,*J*,*J*), Γ= *tridiag*(Z,B,Z) − *sparse*(2,1,−*a* + *b*,*J*,*J*)−*sparse*(*J* − 1,*J*,−*a* − *b*,*J*,*J*), A, B, I, Z are block matrices, expressed as A = *tridiag*(−*a* + *b*,1 + 2*a*,−*a* − *b*), B = *tridiag*(*a* − *b*,1 − 2*a*,*a + b*), where A and B are matrices with (*J* − 1)×(*J* − 1) elements, I = [1]_1×1,_ Z = [0]_1×1_, *a* = *c*/2, *b* = Δ*t*/(4*r_j_*Δ*r*), *c* = Δ*t*/(Δ*r*^2^) with *j* = 1,2,…*J* − 1, *sparse*(*p,q*,*u*,*J*,*J*) = [*x_i_*_,*j*_]*_J_*_×*J*_ means a sparse matrix denoting that, except for *x_p,q_* = *u*, the remaining elements equal zero. The inhomogeneous term in Equation (13) is discretized as

(32) $$\Psi ={\left\{-(r_{2}+r_{1}+S(r_{2}^{2}-r_{1}^{2})/2+BrF_{2}){\left[v_{s,j}\right]}_{1\times J}/F_{1}+S+Br{\left({\left|g_{s,j}\right|}_{1\times J}^{}\right)}^{n-1}{\left[{\left(g_{s,j}\right)}_{1\times J}\right]}^{2}\right\}}^{T}$$

The coefficients $F_{1}=\int_{r_{1}}^{r_{2}}v_{s,j}rdr$ and $F_{2}=\int_{r_{1}}^{r_{2}}{\left({\left|g_{s,j}\right|}_{1\times J}^{}\right)}^{n-1}{\left[{\left(g_{s,j}\right)}_{1\times J}\right]}^{2}rdr$ are computed by using the composite trapezoidal integration method, *g_s_*_,1_ = (−*v_s_*_,3_ + 4*v_s_*_,2_ − 3*v_s_*_,1_)/(2Δ*r*), *g_s_*_,*j*_ = (*v_s_*_,*j*_*_+_*_1_ − *v_s_*_,*j*−1_)/(2Δ*r*), *g_s_*_,*J*_ = (*v_s_*_,*J*−2_ − 4*v_s_*_,*J*−1_ + 3*v_s_*_,*J*_)/(2Δ*r*), with *j* = 2,3,…,*J* − 1. With the numerical temperature *T^1^* obtained, the mean temperature and Nusselt number can be computed from Equations (15) and (16) by using the composite trapezoidal integration method.

**Figure 2:**
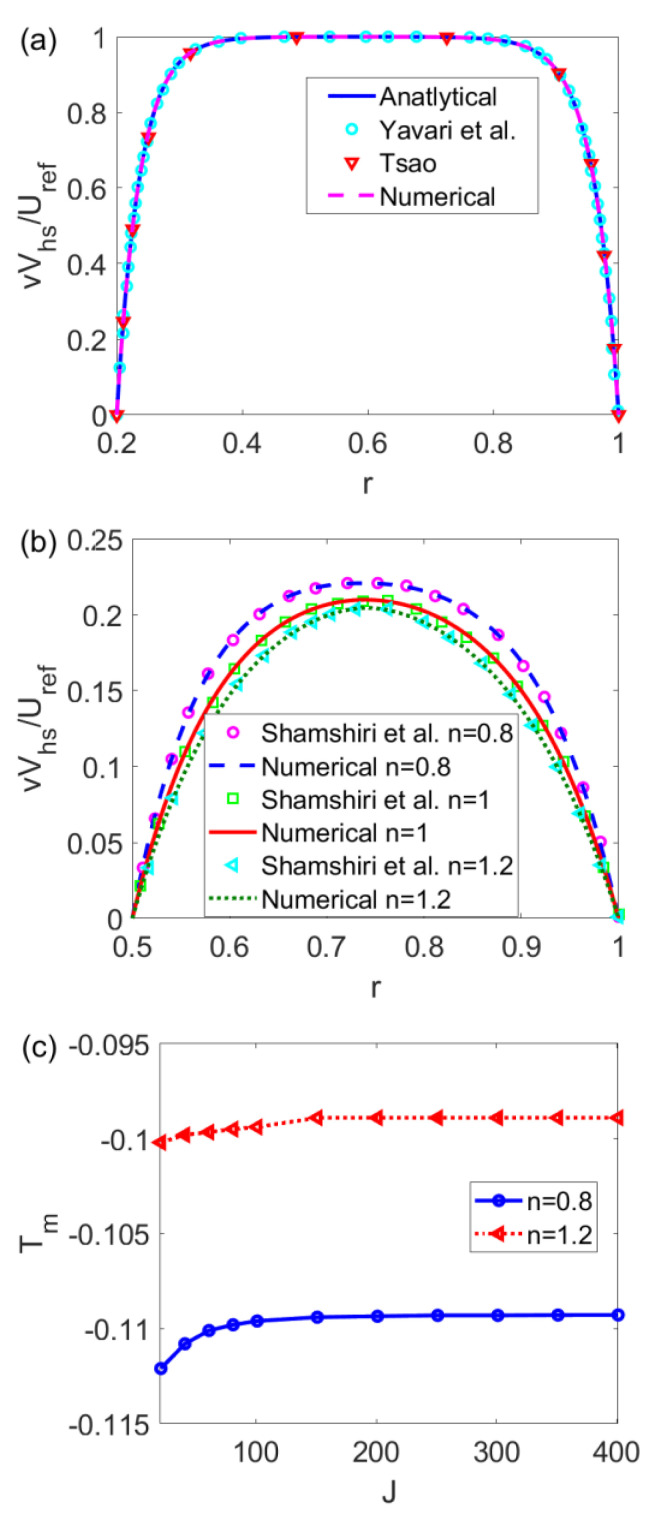
(**a**) The comparison among the analytical velocity, numerical velocity, and existing data from the studies [13,28] without consideration of slip velocity at the channel walls, (**b**) the comparison between numerical velocity and existing data in [30] different *n*, and (**c**) the grid independence study of the mean temperature for shear thinning fluid and shear thickening fluid when *Br* = 0.005, *S* = 2, and *ζ*_1_ = *ζ*_2_ = −1.

In Figure 2a, in the limiting case of a Newtonian fluid, the numerical velocity computed based on Equation (30) is compared with the analytical velocity obtained from Equation (28) and the existing data presented in the study [28] by Yavari et al. and [13] by Tsao. In Figure 2b, for power-law fluids, the numerical velocities computed from Equation (30) are compared with the existing data from [30] by Shamshiri et al. In Figure 2c, the average temperature *T_m_* is numerically computed for different flow behavior indices *n* and grid numbers in terms of space *J*. Irrespective of the value of *n*, when *c* = 0.016 and *J* ≥ 151, the curve of *T_m_* with *J* shows little change, meaning that *J* ≥ 151 is enough to obtain steady numerical solutions. To be economical, the grid number *J* is chosen as 201. Figure 2a,b shows when *J* = 201, the numerical velocities agree well with the analytical velocity or the existing data, meaning that the numerical algorithm is feasible.

## 4. Results

The unsteady hydrodynamics and heat transfer in the electrokinetic slip flow of power-law fluids in a microannulus are investigated by evaluating the unsteady velocity field, flow rate, fully developed temperature field, and Nusselt number at different parameters. The physical parameters take the given values in Nomenclature based on practical uses [4,5,6,39]. To note, ζ_i_^*^ = −0.025V with *i* = 1,2, and the reason for assuming a small zeta potential is to use the Debye–Hückel linearization and the fact that the small zeta potential is physically acceptable [1]. To obtain more realistic predictions, it is essential to present the permissible ranges of governing parameters. Based on the well-established electroosmosis theory of power-law fluid, the flow behavior index *n* ranges from 0.6 to 1.4 [23] and the electrokinetic width *W* ranges from 10 to 100 [4,23]. The order of the Brinkman number *Br* can be of *O*(10^−2^) since the orders of reference velocity, apparent viscosity, and reference radius are identical to that from the studies [7,39]. According to the study [36], the slip lengths *l_i_* range from 0 to 0.1 and the dimensionless pressure gradient ∇*p* is of the order *O*(1), which is chosen as 5.

**Figure 3:**
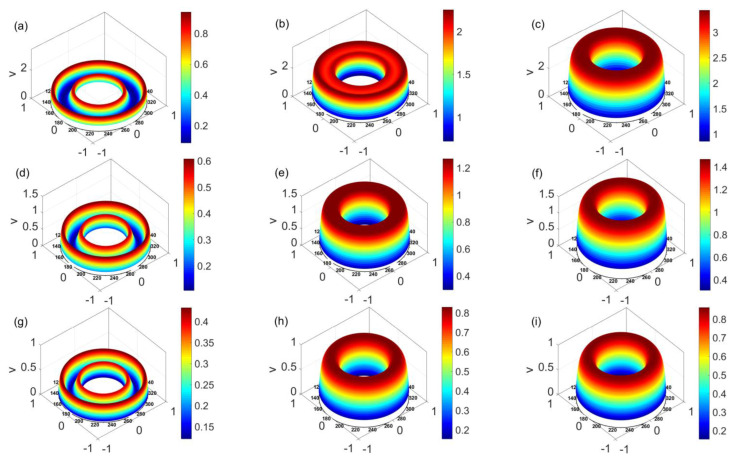
The time evolution of the velocity distribution over a cross-sectioned area of microannulus (**a**) *n* = 0.8, *t* = 0.006, (**b**) *n* = 0.8, *t* = 0.06, (**c**) *n* = 0.8, *t* = 0.6, (**d**) *n* = 1, *t* = 0.006, (**e**) *n* = 1, *t* = 0.06, (**f**) *n* = 1, *t* = 0.6, (**g**) *n* = 1.2, *t* = 0.006, (**h**) *n* = 1.2, *t* = 0.06, and (**i**) *n* = 1.2, *t* = 0.6 when *l*_1_ = *l*_2_ = 0.01 and *W* = 30.

Figure 3 presents the unsteady velocity distribution over a cross-sectioned area of a microannulus at *t* = 0.006, *t* = 0.06, and *t* = 0.6 for the shear thinning, Newtonian, and shear thickening fluids. From Figure 3a,d,g, irrespective of the fluid type, at first the fluid at and near the channel walls is set in motion under the electroosmotic force and slippery effect, which then drags the bulk fluid in the central area forward under the shear stress and pressure gradient, forming the pressure driven electrokinetic slip flow. Figure 3a–c shows that for the shear thinning fluid, as time evolves from 0.006 to 0.6, the velocity grows obviously, and the magnitude of velocity eventually goes beyond 3. In contrast, Figure 3g–i shows that for the shear thickening fluid, from *t* = 0.006 to *t* = 0.06, the velocity distribution evolves, however, it changes little when time lapses from *t* = 0.06 to *t* = 0.6. The comparison among Figure 3a–c,d–f,g–i indicates that as time evolves, the shear thinning fluid attains the greatest velocity which is two times more than that of Newtonian and shear thickening fluids. Moreover, as time goes beyond 0.06, the change in velocity becomes smaller when the fluid changes from a shear thinning to a shear thickening fluid, meaning that the shear thickening fluid achieves the steady state earlier than the shear thinning and Newtonian fluids. In addition, since *l_i_* = 0.01 (*i* = 1, 2), the fluid at the channel walls is not static and slips over the solid walls.

**Figure 4:**
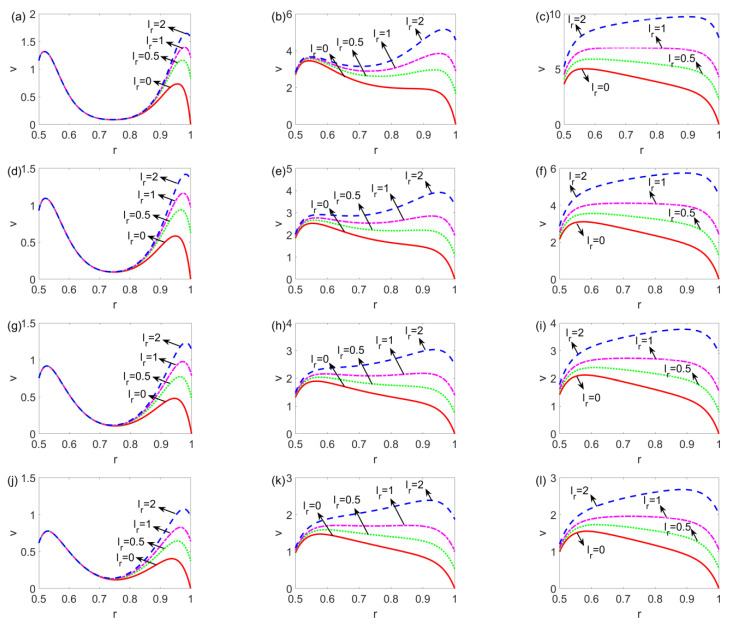

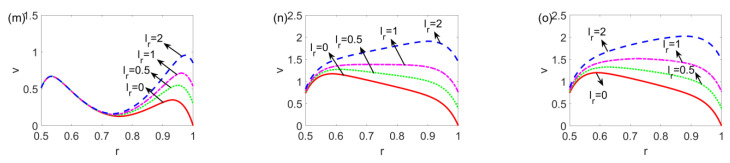
The variation of the velocity profile with a ratio of slip length *l_r_* at different time and flow behavior indices (**a**) *n* = 0.8, *t* = 0.006, (**b**) *n* = 0.8, *t* = 0.06, (**c**) *n* = 0.8, *t* = 0.6, (**d**) *n* = 0.9, *t* = 0.006, (**e**) *n* = 0.9, *t* = 0.06, (**f**) *n* = 0.9, *t* = 0.6, (**g**) *n* = 1, *t* = 0.006, (**h**) *n* = 1, *t* = 0.06, (**i**) *n* = 1, *t* = 0.6, (**j**) *n* = 1.1, *t* = 0.006, (**k**) *n* = 1.1, *t* = 0.06, (**l**) *n* = 1.1, *t* = 0.6, (**m**) *n* = 1.2, *t* = 0.006, (**n**) *n* = 1.2, *t* = 0.06, and (**o**) *n* = 1.2, *t* = 0.6, when *W* = 30, *l*_1_ = 0.05.

As shown in Figure 4, the variation of the velocity profile with slip length ratio *l_r_* (*l*_2_/*l*_1_) at different times is presented for the shear thinning, Newtonian, and shear thickening fluids. In terms of the influence of the slip length ratio, from Figure 4a,d,g,j,m, no matter what type of fluid is considered, since the slip flow is initiated close to the channel walls at first and *l*_1_ is fixed, the flow near the channel walls is accelerated with the increase in *l_r_*. As time evolves, with the bulk liquid set in motion, the influence of the change in the slip length ratio extends from the outer channel wall to the bulk liquid. When *l_r_* = 1, the velocity shows a nearly symmetric profile, while the asymmetric velocity profile is observed when *l_r_* ≠ 1. To note, when *l_r_* = 0, the fluid at the outer channel wall is static all the time, corresponding to the no-slip boundary condition. In addition, different from that under the no-slip condition, the fluid under the slip condition is not subject to resistance at the walls and slips over the solid walls, leading to a higher velocity for the bulk liquid. In terms of the influence of fluid type, namely, *n*, as shown in Figure 4a,d,g,j,m and Figure 4b,e,h,k,n, compared to the shear thinning and Newtonian fluids, the shear thickening fluid in the central area achieves the greatest velocity faster when driven by the surrounding fluid layers, therefore, the flow of shear thickening fluid reaches the steady state earlier, as witnessed in Figure 4k,l,n,o. Furthermore, the comparison among Figure 4c,f,i,l,o shows that the flow of shear thinning fluids tends to exhibit plug-like profiles and that of shear thickening fluids exhibits parabolic profiles.

**Figure 5:**
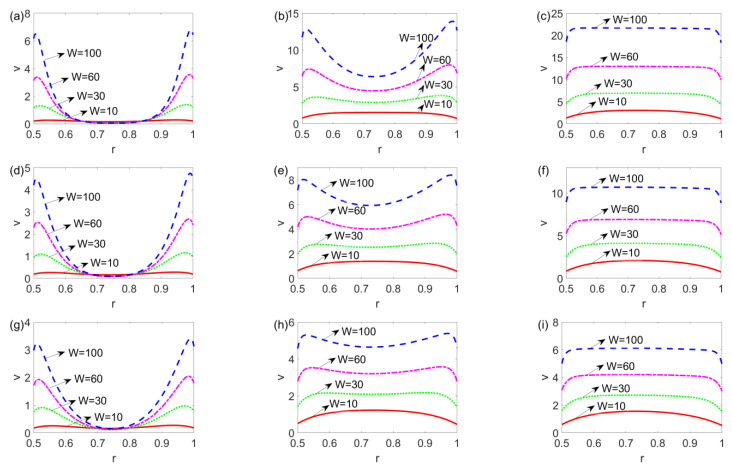

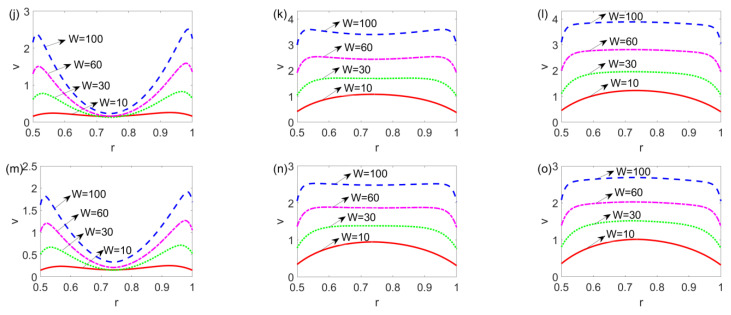
The variation of the velocity distribution with electrokinetic width *W* at a different time and flow behavior indices (**a**) *n* = 0.8, *t* = 0.006, (**b**) *n* = 0.8, *t* = 0.06, (**c**) *n* = 0.8, *t* = 0.6, (**d**) *n* = 0.9, *t* = 0.006, (**e**) *n* = 0.9, *t* = 0.06, (**f**) *n* = 0.9, *t* = 0.6, (**g**) *n* = 1, *t* = 0.006, (**h**) *n* = 1, *t* = 0.06, (**i**) *n* = 1, *t* = 0.6, (**j**) *n* = 1.1, *t* = 0.006, (**k**) *n* = 1.1, *t* = 0.06, (**l**) *n* = 1.1, *t* = 0.6, (**m**) *n* = 1.2, *t* = 0.006, (**n**) *n* = 1.2, *t* = 0.06, and (**o**) *n* = 1.2, *t* = 0.6, when *l*_1_ = *l*_2_ = 0.05.

In Figure 5, the variation of the velocity profile with electrokinetic width *W* at a different time is presented for the shear thinning, Newtonian, and shear thickening fluids. When *t* = 0.006, from Figure 5a,d,g,j,m, since *l_i_* ≠ 0, the fluid at and near the channel walls attains velocity first, increasing with *W* and decreasing with *n*. When the time increases to 0.06, as shown in Figure 5b,e,h,k,n, the fluid near the channel walls drives the fluid in the central area to move forward. The velocity profile of the shear thinning fluid is still developing and the shear thickening fluid flow approaches the developed state. This is especially evident with the greater value of electrokinetic width *W*. From Figure 5c,f,i,l,o, the velocity profile becomes steady, which shows a plug-like pattern when the electrokinetic width changes from 10 to 100 or when the fluid changes from shear thickening to shear thinning. Compared to the Newtonian and shear thickening fluids, the slip flow of a shear thinning fluid is much more sensitive to the change in electrokinetic width *W*.

**Figure 6:**
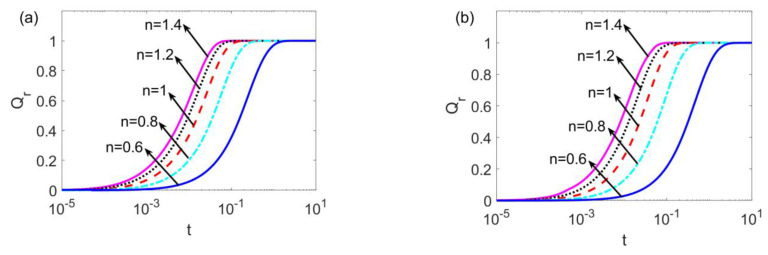
The time evolution of the flow rate at different flow behavior indices *n* when (**a**) *W* = 30, *l*_1_ = *l*_2_ = 0.01, and (**b**) *W* = 30, *l*_1_ = *l*_2_ = 0.05.

Figure 6 illustrates the time evolution of the unsteady flow rate ratio for different flow behavior indices *n* at (a) *l*_1_ = *l*_2_ = 0.01 and (b) *l*_1_ = *l*_2_ = 0.05. The unsteady flow rate ratio is taken as the ratio of the unsteady flow rate to the respective steady flow rate to capture the temporal physical picture of the unsteady slip flow for different types of fluids. As shown in Figure 6a, when *n* ranges from 0.6 to 1.4, the fluid changes from shear thinning to shear thickening, and the slip flow achieves the steady state earlier and earlier. This is due to the fact that the higher apparent viscosity of the shear thickening fluid results in a stronger resistance to the flow and enables the unsteady motion to arrive at the steady state faster. Compared to Figure 6a,b shows that the increase in slip lengths *l_i_* intensifies the driving force near the channel walls; as a result, the unsteady flow needs a longer time to arrive at the steady state.

**Figure 7:**
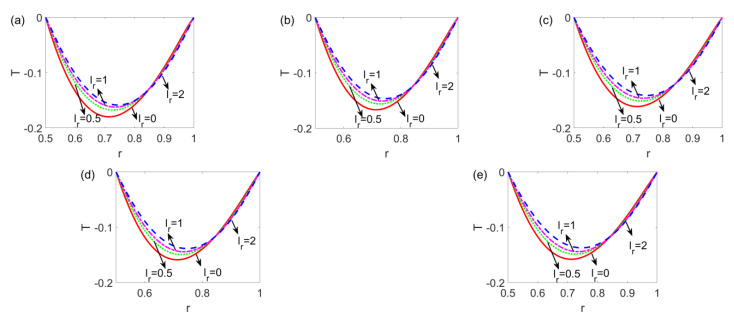
The variation of the temperature profile with a ratio of slip length *l_r_* at different flow behavior indices *n* (**a**) *n* = 0.8, (**b**) *n* = 0.9, (**c**) *n* = 1, (**d**) *n* = 1.1, and (**e**) *n* = 1.2 when *l*_1_ = 0.05, *Br* = 0.005 and *W* = 30.

Figure 7 shows the variation of the temperature profile with slip length ratio *l_r_* for the shear thinning fluid, Newtonian fluid, and shear thickening fluid. Figure 7 shows that no matter what type of fluid is considered, with the increase in the slip length ratio *l_r_*, the left side of the temperature profile augments, and the right side of the temperature profile shows a slight decrement, in the meantime, the minimum value of temperature shifts from left to right. In addition, in the limiting case of the no-slip condition at one channel wall (*l_r_* = 0), the temperature difference between the bulk liquid and channel walls is the widest, decreasing when the slip length ratio is enhanced, meaning that the presence of slip velocity at solid walls promotes the heat transfer of the electrokinetic slip flow. Furthermore, the influence of *n* and that of *l_r_* will not interact with each other.

**Figure 8:**
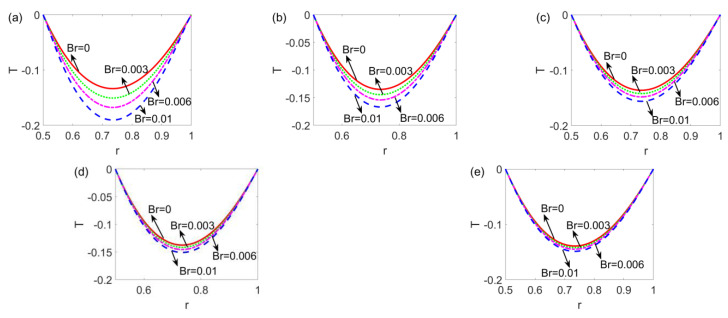
The variation of the temperature profile with Brinkman number *Br* at different flow behavior indices *n* (**a**) *n* = 0.8, (**b**) *n* = 0.9, (**c**) *n* = 1, (**d**) *n* = 1.1, and (**e**) *n* = 1.2 when *l*_1_ = *l*_2_ = 0.05 and *W* = 30.

Figure 8 shows the variation of the temperature profile with the slip length ratio *l_r_* for the shear thinning fluid, Newtonian fluid, and shear thickening fluid. It is obvious that the temperature profile decreases; namely, the temperature difference is widened with the increase in Brinkman number *Br*. The greater Brinkman number implies a stronger viscous dissipation effect which inevitably hinders the heat transfer of the electrokinetic flow; as a result, the widened temperature difference is observed. When the fluid changes from shear thinning to shear thickening, the influence of the Brinkman number Br on the temperature profile is reduced, since the corresponding velocity gradient of electrokinetic flow becomes smaller.

**Figure 9:**
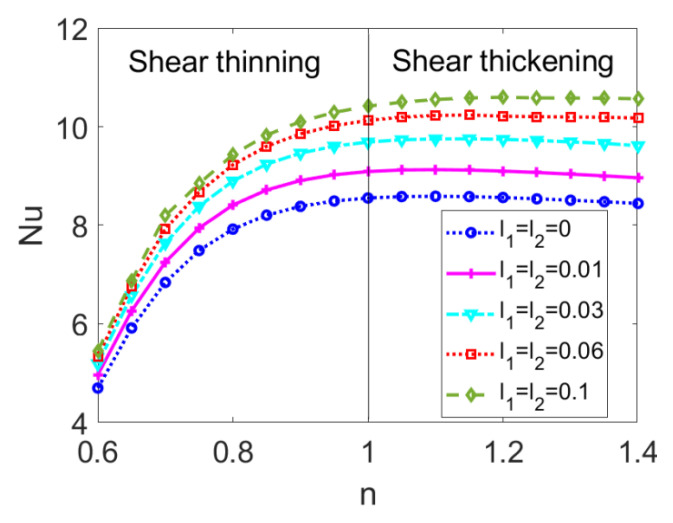
The variation of the Nusselt number *Nu* with flow behavior index *n* when considering different slip lengths when *Br =* 0.005 and *W* = 30.

The variation in the Nusselt number *Nu* with the flow behavior index *n* is presented in Figure 9 for different slip lengths *l_i_*. When considering the shear thinning fluid (*n* < 1), the Nusselt number *Nu* augments with the flow behavior index *n,* and the increasing rate is enhanced with slip lengths *l_i_*. When considering the shear thickening fluid (*n* > 1), the Nusselt number *Nu* shows little change with *n*, though it still increases with *l_i_*, meaning that the influence of *n* on the Nusselt number *Nu* becomes smaller; in other words, *Nu* is not as susceptible as that of the shear thinning fluid to the change in the flow behavior index *n*. The curve of *Nu* with *n* increases as a whole with the slip lengths *l_i_*. Although the flows of both shear thinning and shear thickening fluids are accelerated by the greater slip lengths, the velocity distribution becomes more uniform for shear thickening fluids; in contrast, a wider velocity difference between the channel walls and central region occurs for shear thinning fluids, which partly suppresses the heat transfer of the bulk liquid. Therefore, the increase in *Nu* with slip lengths is especially significant for shear thickening fluids. Consequently, in engineering practice, when driving the shear thickening fluids in the microannulus, the heat transfer performance can be promoted by adjusting the slip length.

## 5. Conclusions

The temporal physical picture of the unsteady pressure-driven electrokinetic slip flow for power-law fluids in a microannulus is provided, based on which, the heat transfer of a steady flow is analyzed for different governing parameters including the flow behavior index (*n*), slip lengths (*l_i_*), electrokinetic width (*W*), and Brinkman number (*Br*). In terms of the temporal hydrodynamical behavior of power-law fluids, the flow is accelerated with the increase in slip lengths and electrokinetic width and the flow of shear thinning fluids is greater and much more sensitive to the change of the above parameters than that of Newtonian and shear thickening fluids. When the fluid changes from shear thinning to shear thickening or the slip lengths are reduced, the flow reaches the steady state earlier. In terms of the thermal behavior of the steady flow, the temperature profile increases with the slip length ratio and flow behavior index, which decreases with the Brinkman number, meaning that the more uniform the velocity distribution, the narrower the temperature difference between the channel walls and bulk liquid, and the more intense the heat transfer performance. Therefore, in practical uses, the slip surface can be reliably engineered to achieve a higher flow rate and promote heat transfer. The analysis above can serve as a theoretical guidance for the design and optimum operation of relevant microfluidic devices.

## Data Availability

The data that support the findings of this study are available from the corresponding author upon reasonable request.

## Nomenclature

*Br*	Brinkman number
*e*	Elementary charge, 1.6 × 10^−19^ C
*E*_0_	Strength of applied electric field, 1 × 10^4^ V·m^−1^
*h*	Convective heat transfer coefficient (W∙m^−2^∙K^−1^)
*k*	Thermal conductivity, 0.618 W·m^−1^·K^−1^
*k_B_*	Boltzmann constant, 1.38 × 10^−23^ J·K^−1^
*l_i_*	Dimensionless slip length
*m*_0_	Dynamic viscosity of Newtonian fluid, 9 × 10^−4^ N·m^−2^·s
*m_n_*	Flow consistency index^,^ 9 × 10^−4^ N·m^−2^·s^n^
*n*	Flow behavior index
*n*_0_	Ion concentration (m^−3^)
*Nu*	Nusselt number
*p*	Dimensionless pressure
*q_s_*	Heat flux (W·m^−2^)
*Q*	Dimensionless flow rate
*r*	Dimensionless radial coordinate
*r_i_*	r_1_ for dimensionless inner radius, r_2_ for dimensionless outer radius
*R*	Reference radius, 1 × 10^−4^ m
*S*	Joule heating parameter
*t*	Dimensionless time
*T_a_*	Absolute temperature, 293 K
*T*	Dimensionless temperature
*v*	Dimensionless velocity
*V_hs_*	Helmholtz–Smoluchowski electroosmotic velocity (m·s^−1^)
*W*	Electrokinetic width of EDL
*z*	Valence of ions, 1
Greek Symbols	
*α*	Dimensionless flow consistency index
*ψ*	Dimensionless electric potential
*ρ_e_*	Net charge density (C·m^−3^)
*ρ*	Density of fluid (kg·m^−3^)
*σ*	Electrical conductivity (W·m^−1^·K^−1^)
*ζ_i_*	Dimensionless zeta potential
*ε*	Fluid permittivity (F·m^−1^)
*η*	Dimensionless power-law nanofluid viscosity
*κ*	Debye–Hückel parameter (m^−1^)
*τ*	Shear stress of power-law fluid (N·m^−2^)
*ρC_p_*	Heat capacity per unit volume (J·m^−3^·K^−1^)
Subscripts	
*i*	i = 1 for inner wall, i = 2 for outer wall
*m*	Mean value
*s*	Steady flow
*w*	Walls
*ν*	Order of Bessel function
*r*	Ratio of the outer wall to the inner wall
Superscripts	
*N*	Newtonian fluid
*	Dimensional version
*ꞌ*	First order derivative

## Appendix A

In Equation (25),

(A1) $$C_{m}=\int_{r_{1}}^{r_{2}}(-W^{2}\psi -\nabla p)rM_{0}(\lambda_{m}r)dr/G_{0}(\lambda_{m})\;=\frac{\nabla p\left[r_{2}L(\lambda_{m},r_{2})-r_{1}L(\lambda_{m},r_{1})\right]}{-\lambda_{m}^{}G_{0}(\lambda_{m})}+\frac{W^{2}\left[r_{2}G(\lambda_{m},r_{2})-r_{1}G(\lambda_{m},r_{1})\right]}{-(W^{2}+\lambda_{m}^{2})G_{0}(\lambda_{m})}$$

(A2) $$G_{0}(\lambda_{m}r)=Z_{m}J_{0}(\lambda_{m}r)-W_{m}Y_{0}(\lambda_{m}r)$$

(A3) $${\tilde{M}}_{0}(\lambda_{m})=Z_{m}^{2}[N_{Z}(\lambda_{m},r_{2})-N_{Z}(\lambda_{m},r_{1})]+W_{m}^{2}[N_{W}(\lambda_{m},r_{2})-N_{W}(\lambda_{m},r_{1})]\;-2Z_{m}^{}W_{m}[N(\lambda_{m},r_{2})-N(\lambda_{m},r_{1})]$$

(A4) $$L(\lambda_{m},r)=Z_{m}J_{1}(\lambda_{m}r)-W_{m}Y_{1}(\lambda_{m}r)$$

(A5) $$G(\lambda_{m},r)=Z_{m}\left[\lambda_{m}J_{1}(\lambda_{m}r)\psi +J_{0}(\lambda_{m}r)\psi^{\prime }\right]-W_{m}\left[\lambda_{m}Y_{1}(\lambda_{m}r)\psi +Y_{0}(\lambda_{m}r)\psi^{\prime }\right]$$

(A6) $$N_{Z}(\lambda_{m},r)=r^{2}[J_{0}^{2}(\lambda_{m}r)+J_{1}^{2}(\lambda_{m}r)]/2$$

(A7) $$N_{W}(\lambda_{m},r)=r^{2}[Y_{0}^{2}(\lambda_{m}r)+Y_{1}^{2}(\lambda_{m}r)]/2$$

(A8) $$N(\lambda_{m},r)=r^{2}[J_{0}(\lambda_{m}r)Y_{0}(\lambda_{m}r)+J_{1}(\lambda_{m}r)Y_{1}(\lambda_{m}r)]/2$$

(A9) $$\psi^{\prime }=AWI_{1}(Wr)-BWK_{1}(Wr)$$

*I*_1_ and *K*_1_ mean the 1-th order Bessel functions of the first kind and second kind, respectively, and the relevant coefficients are

(A10) $$Z_{m}=Y_{0}(\lambda_{m}r_{1})-l_{1}\lambda_{m}Y_{1}(\lambda_{m}r_{1})+Y_{0}(\lambda_{m}r_{2})+l_{2}\lambda_{m}Y_{1}(\lambda_{m}r_{2})$$

(A11) $$W_{m}=J_{0}(\lambda_{m}r_{1})-l_{1}\lambda_{m}J_{1}(\lambda_{m}r_{1})+J_{0}(\lambda_{m}r_{2})+l_{2}\lambda_{m}J_{1}(\lambda_{m}r_{2})$$

where *λ_m_*, (*m* = 1,2,…) are the roots of following transcendental equation

(A12) $$\left[J_{0}(\lambda_{m}r_{2})+l_{2}\lambda_{m}J_{1}(\lambda_{m}r_{2})][Y_{0}(\lambda_{m}r_{1})-l_{1}\lambda_{m}Y_{1}(\lambda_{m}r_{1})\right]\;-[J_{0}(\lambda_{m}r_{1})-l_{1}\lambda_{m}J_{1}(\lambda_{m}r_{1})][Y_{0}(\lambda_{m}r_{2})+l_{2}\lambda_{m}Y_{1}(\lambda_{m}r_{2})]=0$$

with *J_ν_* and *Y_ν_* (*i* = 0,1) denoting the *ν*-th order Bessel functions of the first kind and second kind.

The coefficients in Equation (27) are given as

(A13) $$D=\frac{\zeta_{2}+\nabla pr_{2}^{2}/4+l_{1}[\psi^{\prime }(r_{1})+\nabla pr_{2}^{}/2]-\zeta_{1}-\nabla pr_{1}^{2}/4+l_{2}[\psi^{\prime }(r_{2})+\nabla pr_{2}^{}/2]}{\ln r_{1}-l_{2}/r_{2}-\ln r_{2}-l_{1}/r_{1}}$$

(A14) $$E=l_{1}[\psi^{\prime }(r_{1})+\nabla pr_{1}/2+D/r_{1}]-(\zeta_{1}+\nabla pr_{1}^{2}/4+D\ln r_{1})$$

The coefficients and intermediate functions *P*(*r*) and *Q*_0_(*r*) in Equation (29) are

(A15) $$T_{0}=-P(r_{2})-\left[C_{0}-Q_{0}(r_{1})\right]\ln r_{2}$$

(A16) $$C_{0}=\frac{P(r_{2})-P(r_{1})+Q_{0}(r_{1})\left(\ln r_{1}-\ln r_{2}\right)}{\ln r_{1}+\ln r_{2}}$$

(A17) $$F=\frac{r_{1}+r_{2}+S(r_{2}^{2}-r_{1}^{2})}{P_{0}(r_{2})-P_{0}(r_{1})}$$

(A18) $$P(r)=\frac{F}{W^{2}}\psi +\frac{F\nabla p}{64}r^{4}+\frac{1}{4}(EF-S-DF)r^{2}+\frac{DF}{4}r^{2}\ln r$$

(A19) $$Q_{0}(r)=\frac{F}{W}r\psi^{\prime }+\frac{F\nabla p}{16}r^{4}+\frac{1}{4}(2EF-2S-DF)r^{2}+\frac{DF}{2}r^{2}\ln r$$

in which

(A20) $$P_{0}(r)=\frac{1}{W}r\psi^{\prime }+\frac{\nabla p}{16}r^{4}+\frac{1}{4}(2E-D)r^{2}+\frac{D}{2}r^{2}\ln r$$
